# Association of PADUA and RENAL scores with early perioperative outcomes in large renal tumors managed with robot-assisted partial nephrectomy

**DOI:** 10.3389/fsurg.2026.1801634

**Published:** 2026-04-30

**Authors:** Tunkut Doganca, Boran Aksakal, Osman Acar, Ceyda Buyuker, Ilter Tufek, Ali Riza Kural

**Affiliations:** 1 Department of Urology, Acibadem Taksim Hospital, Istanbul, Türkiye; 2 School of Medicine, Acibadem Mehmet Ali Aydinlar University, Istanbul, Türkiye; 3 Department of Urology, School of Medicine, Acibadem Mehmet Ali Aydinlar University, Istanbul, Türkiye

**Keywords:** PADUA score, perioperative outcomes, RENAL nephrometry score, renal tumor complexity, robot-assisted partial nephrectomy, trifecta, warm ischemia time

## Abstract

**Introduction:**

Tumor complexity scoring supports preoperative planning for robot-assisted partial nephrectomy (RAPN), but evidence in larger tumors is limited. We evaluated whether PADUA and RENAL nephrometry scores are associated with early perioperative outcomes in a stage-focused cohort of pathological pT1b or higher renal tumors treated with multiport RAPN.

**Methods:**

We retrospectively reviewed a prospectively maintained single-center database of multiport RAPN cases (2011–2025). Patients with pathological pT1b–pT2a disease were included. The primary endpoint was a 30-day composite adverse outcome: major complications (Clavien–Dindo ≥3) and/or perioperative transfusion. Secondary endpoints were any complication (Clavien 1–5), transfusion, warm ischemia time (WIT; continuous and >25 min), operative time, length of stay, and trifecta achievement. Associations were tested using logistic regression (penalized models for sparse outcomes), linear regression, and AUC; *β* represents the adjusted change in the outcome per 1-point increase in score.

**Results:**

In total, 109 patients were included (PADUA available in 108; RENAL in 83). Neither PADUA nor RENAL was independently associated with the composite adverse outcome, and discrimination was limited (PADUA AUC 0.583, 95% CI 0.411–0.733; RENAL AUC 0.563, 95% CI 0.347–0.775; *p* = 0.738). Both scores were associated with intraoperative metrics. Each 1-point increase in PADUA correlated with longer WIT (β 1.13 min; 95% CI 0.20–2.07; *p* = 0.019) and operative time (β 4.55 min; 95% CI 0.20–8.90; *p* = 0.043). Each 1-point increase in RENAL correlated with longer WIT (*β* 1.33 min; 95% CI 0.25–2.40; *p* = 0.018) and operative time (β 5.01 min; 95% CI 1.62–8.40; *p* = 0.005). Trifecta achievement was not significantly associated with either score.

**Conclusion:**

In patients with pathological pT1b or higher renal tumors treated with multiport RAPN, PADUA and RENAL scores were not associated with a 30-day composite adverse outcome and had limited discrimination for major morbidity and/or transfusion, but both correlated with WIT and operative time. Larger externally validated cohorts incorporating longitudinal renal function and broader patient-level risk factors are needed to refine preoperative risk assessment in larger renal tumors.

## Introduction

Robot-assisted partial nephrectomy (RAPN) has become a widely adopted approach for the management of localized renal masses and a cornerstone minimally invasive option for nephron-sparing surgery. Contemporary series and comparative studies have shown that RAPN can achieve favorable perioperative outcomes, and in selected settings, its performance is at least comparable to laparoscopic and open partial nephrectomy (1–3).

As surgical indications expand to include larger and anatomically more challenging tumors, accurate preoperative assessment of tumor complexity is increasingly important for surgical planning, patient counseling, and risk stratification. Rather than relying on individual tumor features such as size or location alone, structured scoring systems were developed to summarize key anatomic characteristics into a standardized complexity measure. The PADUA score and the RENAL score, both introduced in 2009, quantify tumor anatomy on the basis of cross-sectional imaging and were proposed to support comparisons across patients and to estimate the risk of perioperative morbidity (4).

Evidence synthesis suggests that nephrometry scores have value in predicting selected perioperative outcomes after nephron-sparing surgery; however, reported performance varies by outcome definition, patient selection, and surgical approach (5). In addition, major guidelines emphasize nephron-sparing strategies for appropriate localized disease and highlight the importance of individualized decision-making, which further underscores the clinical relevance of reliable preoperative risk tools (6, 7).

In this study, we evaluated the relationship between tumor complexity and early perioperative outcomes in a single-center cohort of patients with pathological stage pT1b or higher renal tumors managed with multiport RAPN. Using the PADUA score and the RENAL scores derived from preoperative imaging, we aimed to assess their associations with a 30-day composite adverse outcome (major complications defined as Clavien–Dindo ≥3 and/or perioperative blood transfusion), as well as secondary outcomes, including any complication, blood transfusion, trifecta achievement, warm ischemia time, operative time, and length of hospital stay (4).

## Materials and methods

### Data collection

The study was approved by the Institutional Research Ethics Committee of Acibadem Mehmet Ali Aydinlar University (ATADEK-2026-02/52). A prospectively maintained, single-center database was reviewed and all patients who underwent RAPN between 2011 and 2025 were retrospectively identified. Operations were performed by the same robotic surgery team via da Vinci robotic platform (Intuitive Surgical, Sunnyvale, CA, USA). The robotic urology program started in 2005. To date, the program has performed more than 3,000 robotic urologic procedures (prostate, kidney, and bladder). By the start of the study period (2011), the team had already performed a high volume of robotic urologic procedures, and all cases in this cohort were performed by the same dedicated robotic surgery team. Patient demographics, operative details, perioperative data, and 30-day post-operative outcomes were collected. Tumor anatomical complexity was recorded via RENAL score and PADUA score, which were calculated from preoperative cross-sectional imaging. When needed, missing or unclear entries were checked via electronic medical records.

### Study population

Study population were the patients who underwent multiport RAPN with final pathological stage of pT1b or higher disease. Patients with pT1a tumors and missing data inconclusive to determine primary or secondary outcomes were excluded. PADUA and RENAL nephrometry scores were analyzed in all available cases and paired comparisons were performed.

### Assessment of tumor complexity

Tumor complexity was assessed with preoperative cross-sectional imaging via RENAL score and PADUA score. Both scores were analyzed as continuous variables and grouped by commonly used cutoffs: For RENAL score: Low (≤6), intermediate (7–9), and high (≥10) and for PADUA score: Low (≤7), intermediate (8, 9), and high (≥10). To avoid collinearity, RENAL score and PADUA score were evaluated in separate multivariable models. The individual components and definitions of the PADUA and RENAL nephrometry scoring systems are summarized in Supplementary Table S1.

### Operative technique

All procedures were performed through a transperitoneal approach. Patients were placed in a modified flank position with the operative side up, and the table was flexed to improve exposure. Pneumoperitoneum was created with an open Hasson entry. The colon was mobilized to reach the kidney, and the renal hilum was exposed. The renal artery was then dissected and prepared for clamping.

A laparoscopic or robotic ultrasound probe was used to define the limits of the tumor and evaluate its relationship with the collecting system. The planned excision line was marked around the lesion on the renal surface. The renal artery was clamped with one or two bulldog clamps depending on intraoperative findings. The tumor was removed by resection, enucleation, or a combined technique based on tumor anatomy.

Reconstruction was carried out in layers. The tumor bed was closed with a running 3–0 barbed suture to control bleeding and repair collecting system opening when present. The outer renorrhaphy was completed with 0 polyglactin sutures via sliding-clip technique. After unclamping, the resection site was checked again, and additional sutures or hemostatic measures were used if necessary. Oxidized regenerated cellulose was applied routinely, and other topical agents were used only when needed (8).

### Outcome definition

The primary endpoint was a 30-day composite adverse outcome, defined as the occurrence of major postoperative complications (Clavien–Dindo grade ≥3) and/or perioperative blood transfusion. Postoperative complications within 30 days were graded by using the Clavien–Dindo classification. Perioperative transfusion was administered according to institutional practice in cases of hemoglobin <8 g/dL and/or symptomatic anemia with hemodynamic changes (e.g., hypotension or tachycardia), at the discretion of the surgical and anesthesiology teams. Transfusion was included in the composite endpoint because it represents a clinically meaningful early perioperative adverse event reflecting bleeding severity and perioperative management burden. We acknowledge that transfusion thresholds may vary across institutions; therefore, transfusion is also reported as a separate secondary endpoint.

The secondary endpoints were as follows: (1) any complication within 30 days (Clavien 1–5; yes/no); (2) blood transfusion as an individual outcome (yes/no); (3) trifecta achievement (negative surgical margin, no postoperative complications, and WIT ≤25 min); (4) warm ischemia time (WIT) >25 min [WIT analyzed as a continuous variable (minutes) and as a binary variable]; (5) operative time (minutes, as recorded in the institutional database); and (6) length of hospital stay (LOS; days).

### Statistical analysis

Continuous variables are summarized as medians (interquartile ranges), and categorical variables are summarized as counts (percentages). Comparisons across RENAL and PADUA complexity categories were performed via the Kruskal–Wallis test for continuous variables and the chi-square test or Fisher exact test for categorical variables.

For the primary endpoint, separate multivariable logistic regression models were built for the RENAL score and the PADUA score to estimate odds ratios with 95% confidence intervals. Because the event rate was expected to be low, model complexity was limited; penalized logistic regression (Firth method) was used if model instability or separation was observed. Discrimination was assessed with receiver operating characteristic analysis and the area under the curve (AUC), and the AUCs were compared via the DeLong method when applicable. Spearman rank correlation coefficients were used to assess correlations between nephrometry scores and continuous perioperative metrics. A two-sided *p* value < 0.05 was considered statistically significant. All analyses were performed by using R (R Foundation for Statistical Computing, Vienna, Austria).

## Results

### Patient cohort and missing data

A total of 109 patients with pathological stage pT1b (*n* = 106) and pT2a (*n* = 3) disease were enrolled. The composite adverse outcome (Clavien–Dindo ≥ 3 and/or perioperative transfusion) occurred in 10 patients (9.2%). PADUA score was available for 108 patients (low: *n* = 12; intermediate: *n* = 31; high: *n* = 65), whereas RENAL score was available for 83 patients (low: *n* = 14; intermediate: *n* = 53; high: *n* = 16). RENAL scores were missing primarily due to incomplete documentation in earlier years of the cohort, when nephrometry scoring was not a constituent of our structured database. The baseline characteristics and perioperative outcomes of the PADUA and RENAL nephrometry score complexity groups are summarized in Tables 1, 2, respectively. Major 30-day complications (Clavien–Dindo ≥ 3) are further detailed by type in Supplementary Table S2.

**Table 1:** Baseline demographic, clinical, and perioperative characteristics of patients with pT1b-pT2a renal tumors undergoing robot-assisted partial nephrectomy according to PADUA score categories.

Variable	Low (≤7)	Intermediate (8–9)	High (≥10)	*P*
	(*N* = 12)	(*N* = 31)	(*N* = 65)	
Male (proportion)	8 (0.67)	25 (0.81)	46 (0.71)	0.514
Age (year)	51.5	50.0	49.0	0.460
	(43.0–68.2)	(43.0–66.0)	(39.0–63.0)	
ASA ≥3, *n* (proportion)	2 (0.17)	2 (0.06)	1 (0.02)	0.061
BMI (kg/m²)	27.5	28.0	28.0	0.852
	(24.8–32.0)	(26.0–30.0)	(26.0–33.0)	
Tumor size (mm)	44.0	50.0	50.0	0.084
	(42.0–47.2)	(45.0–57.0)	(45.0–55.0)	
Warm ischemia time (min)	19.5	20.0	21.0	0.029
	(6.8–21.0)	(18.5–21.0)	(20.0–24.0)	
Operative time (min)	103.5	110.0	120.0	0.184
	(80.2–110.5)	(89.0–129.0)	(93.0–135.0)	
Estimated blood loss (mL)	175.0	200.0	200.0	0.454
	(100.0–300.0)	(100.0–400.0)	(100.0–400.0)	
Length of stay (days)	4.0	4.0	4.0	0.771
	(3.0–5.0)	(4.0–4.0)	(4.0–4.0)	
Any complication, *n* (proportion)	0 (0.00)	3 (0.10)	8 (0.12)	0.430
Clavien ≥3, *n* (proportion)	0 (0.00)	2 (0.06)	6 (0.09)	0.518
Blood transfusion, *n* (proportion)	0 (0.00)	3 (0.10)	3 (0.05)	0.403
Composite adverse outcome (proportion)	0 (0.00)	3 (0.10)	7 (0.11)	0.495
WIT >25 min, *n* (proportion)	0 (0.00)	1 (0.03)	9 (0.14)	0.123
Positive surgical margin, *n* (proportion)	1 (0.09)	0 (0.00)	1 (0.02)	0.156
Trifecta achievement, *n* (proportion)	10 (0.91)	27 (0.87)	48 (0.75)	0.245

Patient demographics, tumor characteristics, and perioperative outcomes are summarized across PADUA risk categories (low, intermediate, high). Continuous variables are presented as median (IQR) and categorical variables as *n* (%). *P* values refer to between-group comparisons.

**Table 2:** Baseline characteristics and outcomes according to RENAL complexity categories.

Variable	Low (≤6)	Intermediate (7–9)	High (≥10)	*P*
	(*N* = 14)	(*N* = 53)	(*N* = 16)	
Male (proportion)	9 (0.64)	41 (0.77)	10 (0.62)	0.388
Age (year)	52.5	50.0	49.0	0.576
	(43.2–69.0)	(40.0–64.0)	(33.5–53.0)	
ASA ≥3, *n* (proportion)	1 (0.07)	2 (0.04)	0 (0.00)	0.576
BMI (kg/m^2^)	29.0	28.0	28.0	0.930
	(27.0–30.0)	(25.0–32.0)	(26.0–30.0)	
Tumor size (mm)	45.0	50.0	50.0	0.445
	(44.0–57.0)	(45.0–55.0)	(46.0–60.5)	
Warm ischemia time (min)	17.5	20.0	21.0	0.042
	(2.2–20.0)	(19.0–22.0)	(19.0–24.2)	
Operative time (min)	92.5	100.0	121.0	0.010
	(80.8–110.0)	(90.0–120.0)	(108.8–134.2)	
Estimated blood loss (mL)	200.0	200.0	325.0	0.312
	(100.0–375.0)	(100.0–400.0)	(187.5–500.0)	
Length of stay (days)	4.0	4.0	4.0	0.889
	(3.2–4.8)	(4.0–4.0)	(3.8–4.2)	
Any complication, *n* (proportion)	1 (0.07)	4 (0.08)	3 (0.19)	0.388
Clavien ≥3, *n* (proportion)	0 (0.00)	4 (0.08)	2 (0.12)	0.414
Blood transfusion, *n* (proportion)	1 (0.07)	4 (0.08)	0 (0.00)	0.529
Composite adverse outcome, *n* (proportion)	1 (0.07)	5 (0.09)	2 (0.12)	0.881
WIT >25 min, *n* (proportion)	0 (0.00)	4 (0.08)	1 (0.06)	0.572
Positive surgical margin, *n* (proportion)	1 (0.07)	1 (0.02)	0 (0.00)	0.415
Trifecta achievement, *n* (proportion)	12 (0.86)	44 (0.85)	12 (0.75)	0.640

Patient demographics, tumor characteristics, and perioperative outcomes are summarized across RENAL score categories (low, intermediate, high). Continuous variables are presented as median (IQR) and categorical variables as *n* (%). *P* values refer to between-group comparisons.

### Outcomes by PADUA score complexity group

The warm ischemia time differed among the PADUA groups and increased with increasing complexity (*p* = 0.029). Operative time, estimated blood loss, and length of stay were comparable between the PADUA groups (all *p* > 0.05; Table 1).

For the 30-day outcomes, the rates of any complication, major complications (Clavien–Dindo ≥ 3), blood transfusion, and composite adverse outcomes did not differ significantly among the PADUA groups (all *p* > 0.05). The rates of positive surgical margins and trifecta achievement were also similar between the PADUA groups (Table 1).

### Outcomes by RENAL score complexity groups

Warm ischemia time (*p* = 0.042) and operative time (*p* = 0.010) increased with increasing RENAL scores. Estimated blood loss and length of stay did not differ between the RENAL score groups (*p* > 0.05; Table 2).

The rates of any complication, major complications, transfusion, and composite adverse outcomes were comparable among RENAL score groups (all *p* > 0.05). The rates of positive surgical margins and trifecta achievement were also similar (Table 2).

### Primary endpoint: logistic regression for composite adverse outcomes

Using Firth penalized logistic regression to account for the low event rate, neither the PADUA score nor the RENAL score was associated with the composite adverse outcome in univariable analyses (both *p* > 0.05) (Table 3). Because the number of composite events was small, we kept the multivariable models simple to avoid overfitting; univariable associations for key clinical covariates (age, ASA, BMI, and tumor size) are shown in Table 3. After adjustment for age and ASA score, the PADUA score remained non-significant (OR 1.204, 95% CI 0.810–1.790; *p* = 0.358), and the RENAL score was also not an independent predictor in the corresponding multivariable model (OR 1.183, 95% CI 0.760–1.841; *p* = 0.456) (Table 4).

**Table 3:** Univariable logistic regression analysis for composite adverse outcome.

	Univariate analysis			
Variable	*N*	*P*	OR	95% CI for OR
Age (per year)	109	0.167	1.032	0.987–1.080
PADUA Score (per unit)	108	0.391	1.198	0.793–1.811
RENAL Score (per unit)	83	0.642	1.114	0.708–1.753
ASA (per unit)	109	0.581	1.386	0.435–4.415
BMI (per kg/m2)	109	0.542	0.959	0.839–1.096
Tumor size (per mm)	109	0.645	1.015	0.954–1.079
Male gender	109	0.314	2.524	0.416–15.326

Univariable logistic regression evaluating the association of PADUA score and RENAL score with the primary endpoint (30-day composite adverse outcome: Clavien–Dindo ≥ 3 and/or perioperative blood transfusion). Results are reported as odds ratios (OR) with 95% confidence intervals (CI).

**Table 4:** Multivariable logistic regression analysis for composite adverse outcome.

MULTIVARIABLE ANALYSIS—MODEL A: PADUA (*n* = 108, events = 10)			
Variable	P	OR	95% CI for OR
PADUA Score (per unit)	0.358	1.204	0.810–1.790
Age (per year)	0.196	1.033	0.983–1.086
ASA (per unit)	0.950	0.958	0.247–3.711

MULTIVARIABLE ANALYSIS—MODEL B: RENAL (*n* = 83, events = 8)			
Variable	P	OR	95% CI for OR
RENAL Score (per unit)	0.456	1.183	0.760–1.841
Age (per year)	0.203	1.037	0.981–1.097
ASA (per unit)	0.762	1.276	0.263–6.184

Multivariable logistic regression models evaluating the association of PADUA score and RENAL score with the primary endpoint (30-day composite adverse outcome: Clavien–Dindo ≥ 3 and/or perioperative blood transfusion). Results are reported as odds ratios (OR) with 95% confidence intervals (CI). Models were adjusted for the covariates specified in the Methods.

### Discrimination: PADUA vs. RENAL

The discriminative performance for predicting the composite adverse outcome was limited for both scoring systems. The AUC was 0.583 for the PADUA score (95% CI 0.411–0.733) and 0.563 for the RENAL score (95% CI 0.347–0.775). Among patients with both scores available (*n* = 83; 8 composite events), the paired AUC comparison via the DeLong test revealed no significant difference between the two (*p* = 0.738) (Table 5).

**Table 5:** Comparison of discriminative performance of PADUA and RENAL scores for composite adverse outcome.

Model	*n*	Events	AUC	95% CI	*P*
PADUA Score	108	10	0.583	0.411–0.733	-
RENAL Score	83	8	0.563	0.347–0.775	-
Paired Comparison (*n* = 83, events = 8)					
PADUA Score			0.543		
RENAL Score			0.563		
Difference (PADUA—RENAL)			−0.020		0.738

Discrimination performance for the primary endpoint is shown as area under the receiver operating characteristic curve (AUC) with 95% CI for PADUA and RENAL nephrometry scores. Comparative testing between AUCs is reported as specified in the methods.

### Secondary analyses

In penalized logistic regression models for binary secondary endpoints, neither the PADUA score nor the RENAL score was significantly associated with any complication or transfusion (all *p* > 0.05). Associations with prolonged ischemia (WIT > 25 min) were directionally positive but did not reach statistical significance (PADUA *p* = 0.098; RENAL *p* = 0.058). Trifecta failure also showed no significant association with either scoring system (Table 6A).

**Table 6:** Association of PADUA and RENAL scores with secondary outcomes.

A. Binary Outcomes (Firth Penalized Logistic Regression)						
Outcome	Score	*n*	Events	OR	95% CI	*P*
Any Complication	PADUA	108	11	1.304	0.866–1.964	0.204
	RENAL	83	8	1.252	0.776–2.020	0.356
Blood Transfusion	PADUA	108	6	0.990	0.609–1.610	0.968
	RENAL	83	5	0.844	0.511–1.392	0.506
WIT (>25 min)	PADUA	108	10	1.456	0.932–2.273	0.098
	RENAL	83	5	2.022	0.977–4.184	0.058
Trifecta Failure	PADUA	106	21	1.321	0.957–1.823	0.091
	RENAL	82	14	1.314	0.888–1.946	0.172

B. Continuous Outcomes (Linear Regression)						
Outcome	Score	*n*	Beta (95% CI)		*P*	R²
WIT (min)	PADUA	108	1.13 (0.20–2.07)		0.019*	0.050
	RENAL	83	1.33 (0.25–2.40)		0.018*	0.067
Operative Time (min)	PADUA	108	4.55 (0.20–8.90)		0.043*	0.038
	RENAL	83	5.01 (1.62–8.40)		0.005*	0.094

C. Spearman Correlations						
Outcome	PADUA	RENAL				
WIT	*p* = 0.008*	*p* = 0.010*				
Operative Time	*p* = 0.025*	*p* = 0.006*				

Panel A and Panel B show regression analyses for secondary outcomes by PADUA score and RENAL score [logistic regression for binary outcomes reported as OR (95% CI); linear regression for continuous outcomes reported as β (95% CI) per 1-point increase]. Panel C shows Spearman correlations (*ρ*) between nephrometry scores and continuous perioperative variables. Secondary outcomes include any complication, transfusion, warm ischemia time (continuous and >25 min), operative time, and length of stay.

* indicates statistical significance (*p* < 0.05).

In the linear regression analyses, both scoring systems were significantly associated with continuous operative metrics. Each 1-point increase in the PADUA score was associated with a longer WIT (β 1.13 min; 95% CI 0.20–2.07; *p* = 0.019) and longer operative time (β 4.55 min; 95% CI 0.20–8.90; *p* = 0.043). Each 1-point increase in the RENAL score was associated with a longer WIT (β 1.33 min; 95% CI 0.25–2.40; *p* = 0.018) and longer operative time (β 5.01 min; 95% CI 1.62–8.40; *p* = 0.005) (Table 6B). Spearman correlation analyses were consistent with these findings for both WIT and operative time (Table 6C).

## Discussion

As RAPN has increasingly been applied beyond small, exophytic renal masses toward larger and anatomically demanding tumors, the ability to quantify surgical complexity preoperatively has become central to operative planning, patient counseling, and benchmarking of outcomes (1–4). Since nephrometry systems have been evaluated across heterogeneous cohorts, stage-focused evidence in larger tumors remains relatively limited and complexity-related trade-offs often become more pronounced as tumor size and anatomic challenges increase (4, 5). In this setting, our study contributes stage-focused data by evaluating pT1b or higher pathological stage tumors treated with RAPNs and assessed whether PADUA and RENAL nephrometry score align with early adverse outcomes (6, 7).

In this stage-focused multiport RAPN for a large renal tumor cohort, neither the PADUA score nor the RENAL score was independently associated with the 30-day composite adverse outcome (major complications, Clavien–Dindo ≥ 3, and/or perioperative blood transfusion), and overall discrimination for this endpoint was limited. In contrast, both scoring systems were consistently associated with intraoperative metrics, with increasing complexity linked to longer warm ischemia times and longer operative times.

The published evidence regarding complications is mixed. A systematic review and meta-analysis by Veccia et al. suggested that nephrometry scores often correlate with complications after nephron-sparing surgery, but predictive performance varies across endpoints, study designs, and surgical approaches (9). In large RAPN cohorts, stronger signals, including associations of PADUA and RENAL nephrometry scores with higher-grade complications and other perioperative outcomes, have been reported (10, 11). External validation studies for RENAL score have also shown associations with perioperative outcomes after partial nephrectomy, although effect sizes and the best-predicted endpoints differ between cohorts (12). However, several single-center studies have reported that these scores predict ischemia time and operative time more consistently than they predict major complications (13–15). Our results are compatible with these reports.

In contrast, associations between nephrometry scores and technical intraoperative metrics have been more consistent. Higher RENAL scores have been linked to longer warm ischemia times and an increased likelihood of collecting system entry in minimally invasive partial nephrectomy (16). Multicenter RAPN series have similarly shown that higher PADUA and RENAL scores are associated with prolonged ischemia and longer procedural times, supporting the concept that these systems capture reconstructive and exposure demands (10, 11, 17). Alternative anatomic scoring approaches and simplified systems such as SPARE have also been proposed and evaluated to summarize tumor anatomy and surgical complexity in partial nephrectomy (18–20).

Warm ischemia is clinically relevant beyond operative efficiency because longer ischemia has been linked to worse postoperative renal functional outcomes, supporting the principle that minimizing clamp time remains important in nephron-sparing surgery (21–23). RAPN-specific functional analyses and broader evidence syntheses suggest that the functional impact of ischemia depends on the baseline renal reserve and operative context and that the proposed thresholds vary across studies (24, 25). Accordingly, the effect sizes observed in our cohort; approximately 1–1.3 min longer warm ischemia time and 4.5–5 min longer operative time for 1-point increase in each nephrometry score; likely reflect increasing technical workload and ischemic exposure, with potential relevance in patients at higher functional risk (21–25). Early postoperative eGFR may be variable, and longitudinal follow-up provides a more stable assessment of renal functional recovery. (i.e., at postop 3–6 month) (26). Given the scope of this study, we used warm ischemia time as an early perioperative surrogate associated with renal functional preservation (27).

Several factors may explain consistent associations with intraoperative metrics, but limited discrimination for our primary composite adverse outcomes. First, our primary endpoint emphasized major complications and transfusion within 30 days. These events are influenced not only by tumor anatomy, but also by patient factors and the perioperative context, including anticoagulation exposure and institutional transfusion thresholds (9, 10, 28). Second, major morbidity and transfusion rates may be relatively low in dedicated RAPN programs, which reduces the statistical power and compresses between-group differences. Third, anatomy-based nephrometry does not account for nontumor contributors to surgical difficulty. Imaging-based tools such as the MAP score were developed to predict adherent perinephric fat and have been associated with technical difficulty and outcomes in RAPN and partial nephrectomy cohorts, suggesting that combined anatomy and patient factor models may improve prediction (29, 30).

This study has several limitations. First, retrospective single-center design introduces selection bias and residual confounding factors. Second, event counts for major complications and transfusions were limited, limiting the power of multivariable modeling. With only 10 composite adverse events, the study may be underpowered to detect modest associations between nephrometry scores and major morbidity and/or transfusion. Therefore, non-significant findings should not be interpreted as definitive absence of an effect. Third, follow-up focused on early perioperative outcomes and WIT was evaluated to predict early renal function. Fourth, nephrometry score availability differed between systems. Since RENAL scores were not available for all patients, missingness may not be completely random and could introduce bias, and it also reduced the sample size for RENAL score-based and paired analyses. Fifth, individual component data for the PADUA and RENAL scores were not available; therefore, component-level association analyses could not be performed. Additionally, there were few pT2a cases (*n* = 3), thus, the findings largely reflect pT1b disease. Finally, stage restriction was based on pathological staging, which may differ from preoperative clinical staging, and the cohort reflects the outcomes of a dedicated RAPN team, which may limit generalizability to lower-volume settings.

## Conclusion

To evaluate how the PADUA score and the RENAL scores relate to perioperative outcomes in larger renal tumors, RAPN patients with pathological stage pT1b–pT2a disease were specifically evaluated. In this stage-focused cohort, neither score was independently associated with a 30-day composite adverse outcome defined by major complications (Clavien–Dindo ≥ 3) and/or perioperative transfusion, and discrimination for this endpoint was limited. In contrast, both scores were consistently associated with intraoperative metrics, with each 1-point increase corresponding to longer warm ischemia time and longer operative time, suggesting that these systems primarily reflect technical complexity rather than early major morbidity in this setting. Larger, externally validated studies incorporating longitudinal renal functional outcomes and broader patient-level risk factors are needed to refine preoperative risk assessment for larger tumors undergoing nephron-sparing surgery.

## Data Availability

The raw data supporting the conclusions of this article will be made available by the authors upon reasonable request.

## References

[1] Lee CU Alabbasi M Chung JH Kang M Seo SI. How far has robot-assisted partial nephrectomy reached? Investig Clin Urol. (2023) 64(5):435–47. 10.4111/icu.20230121 37668199 PMC10482664

[2] Shiroki R Fukami N Fukaya K Kusaka M Natsume T Ichihara T Robot-assisted partial nephrectomy: superiority over laparoscopic partial nephrectomy. Int J Urol. (2016) 23(2):122–31. 10.1111/iju.13001 26585191

[3] Grivas N Kalampokis N Larcher A Tyritzis S Rha KH Ficarra V Robot-assisted versus open partial nephrectomy: comparison of outcomes. A systematic review. Minerva Urol Nefrol. (2019) 71(2):113–20. 10.23736/S0393-2249.19.03391-5 30895768

[4] Campbell SC Uzzo RG Karam JA Chang SS Clark PE Souter L Renal mass and localized renal cancer: evaluation, management, and follow-up: AUA guideline (parts I and II). J Urol. (2021) 206(2):199–218. 10.1097/JU.0000000000001911 34115547

[5] Ficarra V Novara G Secco S Macchi V Porzionato A De Caro R Preoperative aspects and dimensions used for an anatomical (PADUA) classification of renal tumors in patients who are candidates for nephron-sparing surgery. Eur Urol. (2009) 56(5):786–93. 10.1016/j.eururo.2009.07.040 19665284

[6] Kutikov A Uzzo RG. The R.E.N.A.L. nephrometry score: a comprehensive standardized system for quantitating renal tumor size, location and depth. J Urol. (2009) 182(3):844–53. 10.1016/j.juro.2009.05.035 19616235

[7] Long JA Arnoux V Fiard G Autorino R Descotes JL Rambeaud JJ External validation of the RENAL nephrometry score in renal tumors treated by partial nephrectomy. BJU Int. (2013) 111(2):233–9. 10.1111/j.1464-410X.2012.11339.x 22788546

[8] Tufek I Mourmouris P Doganca T Obek C Argun OB Tuna MB Robot-assisted partial nephrectomy for T1b tumors: strict trifecta outcomes. JSLS. (2017) 21(1):e2016. 10.4293/JSLS.2016.00113 PMC5357684 28352149

[9] Rosevear HM Gellhaus PT Lightfoot AJ Kresowik TP Joudi FN Tracy CR. Utility of the RENAL nephrometry scoring system in the real world: predicting surgeon operative preference and complication risk. BJU Int. (2012) 109(5):700–5. 10.1111/j.1464-410X.2011.10452.x 21777362

[10] Veccia A Antonelli A Uzzo RG Novara G Kutikov A Ficarra V Predictive value of nephrometry scores in nephron-sparing surgery: a systematic review and meta-analysis. Eur Urol Focus. (2020) 6(3):490–504. 10.1016/j.euf.2019.11.004 31776071

[11] Schiavina R Novara G Borghesi M Ficarra V Ahlawat R Moon DA PADUA And RENAL nephrometry scores correlate with perioperative outcomes of robot-assisted partial nephrectomy: analysis of the vattikuti GQI-RUS database. BJU Int. (2017) 119(3):456–63. 10.1111/bju.13628 27528265

[12] Zhang ZY Tang Q Li XS Zhang Q Mayer WA Wu JY Clinical analysis of the PADUA and the RENAL scoring systems for renal neoplasms: a retrospective study of 245 patients undergoing laparoscopic partial nephrectomy. Int J Urol. (2014) 21(1):40–4. 10.1111/iju.12192 23675903

[13] Ellison JS Montgomery JS Hafez KS Wolf JS Jr Daignault S Miller DC Association of RENAL nephrometry score with outcomes of minimally invasive partial nephrectomy. Int J Urol. (2013) 20(6):564–70. 10.1111/j.1442-2042.2012.03222.x 23126585

[14] Borghesi M Schiavina R Gan M Novara G Mottrie A Ficarra V. Expanding utilization of robotic partial nephrectomy for clinical T1b and complex T1a renal masses. World J Urol. (2013) 31(3):499–504. 10.1007/s00345-013-1095-2 23645411

[15] Buffi NM Saita A Lughezzani G Porter J Dell'Oglio P Amparore D Robot-assisted partial nephrectomy for complex (PADUA score >=10) tumors: techniques and results from a multicenter experience at four high-volume centers. Eur Urol. (2020) 77(1):95–100. 10.1016/j.eururo.2019.03.006 30898407

[16] Casale P Lughezzani G Buffi N Larcher A Porter J Mottrie A. Evolution of robot-assisted partial nephrectomy: techniques and outcomes from the transatlantic robotic nephron-sparing surgery study group. Eur Urol. (2019) 76(2):222–7. 10.1016/j.eururo.2018.11.038 30527786

[17] Okhunov Z Rais-Bahrami S George AK Waingankar N Duty B Okeke Z The comparison of three renal tumor scoring systems: c-index, PADUA and RENAL nephrometry scores. J Endourol. (2011) 25(12):1921–4. 10.1089/end.2011.0301 21905850

[18] Ficarra V Porpiglia F Crestani A Novara G Bravi CA De Nunzio C The simplified PADUA REnal (SPARE) nephrometry system: a novel classification of parenchymal renal tumors suitable for partial nephrectomy. BJU Int. (2019) 124(4):621–8. 10.1111/bju.14772 30963680

[19] Rosiello G Larcher A Fallara G Basile G Cignoli D Colandrea G The impact of intraoperative bleeding on the risk of chronic kidney disease after nephron-sparing surgery. World J Urol. (2021) 39(8):2925–32. 10.1007/s00345-020-03504-5 33123741

[20] Weprin S Falagario U Veccia A Nandanan N Emerson D Ovanez C Simplified PADUA renal (SPARE) nephrometry scoring system: external validation, interobserver variability, and comparison with RENAL and PADUA in a single-center robotic partial nephrectomy series. Eur Urol Focus. (2021) 7(3):591–7. 10.1016/j.euf.2020.05.016 32591285

[21] Bertolo R Garisto J Dagenais J Sagalovich D Stein R Fareed K Transperitoneal robot-assisted partial nephrectomy with minimum follow-up of 5 years: oncological and functional outcomes from a single institution. Eur Urol Oncol. (2019) 2(2):207–13. 10.1016/j.euo.2018.06.012 31017098

[22] Porpiglia F Fiori C Bertolo R Morra I Russo R Piccoli G Long-term functional evaluation of the treated kidney in a prospective series of patients who underwent laparoscopic partial nephrectomy for small renal tumors. Eur Urol. (2012) 62(1):130–5. 10.1016/j.eururo.2012.02.001 22349568

[23] Mir MC Ercole C Takagi T Zhang Z Velet L Remer EM Decline in renal function after partial nephrectomy: etiology and prevention. J Urol. (2015) 193(6):1889–98. 10.1016/j.juro.2015.01.093 25637858

[24] Crockett MG Giona S Whiting D Yossepowitch O Porpiglia F Rogers CG Nephrometry scores: validation of three systems for perioperative outcomes in retroperitoneal robotic partial nephrectomy. BJU Int. (2020) 126(6):e73–81. 10.1111/bju.15262

[25] Carbonara U Simone G Minervini A Gallioli A De Naeyer G Mottrie A Outcomes of robot-assisted partial nephrectomy for completely endophytic renal tumors: a multicenter analysis. Eur J Surg Oncol. (2021) 47(2):432–9. 10.1016/j.ejso.2020.08.012 32868149

[26] Nakamura M Kameyama S Tsuru I Izumi T Ono A Teshima T Predictors of renal function deterioration at one year after off-clamp non-renorrhaphy partial nephrectomy. PLoS One. (2024) 19(5):e0303104. 10.1371/journal.pone.0303104 38739585 PMC11090305

[27] Li S Guo Z Li Y Chan FL Wang S Gu C. The impact of warm ischemia time on short-term renal function after partial nephrectomy: a systematic review and meta-analysis. BMC Urol. (2025) 25(1):121. 10.1186/s12894-025-01803-w 40361128 PMC12070501

[28] Harris KT Ball MW Gorin MA Curtiss KM Pierorazio PM Allaf ME. Transperitoneal robot-assisted partial nephrectomy: a comparison of posterior and anterior renal masses. J Endourol. (2014) 28(6):655–9. 10.1089/end.2013.0608 24422597

[29] Paulucci DJ Whalen MJ Badani KK. Analysis of the transperitoneal approach to robot-assisted laparoscopic partial nephrectomy for the treatment of anterior and posterior renal masses. J Laparoendosc Adv Surg Tech A. (2015) 25(11):880–5. 10.1089/lap.2015.0308 26368056

[30] Marconi L Challacombe B. Robotic partial nephrectomy for posterior renal tumors: retro or transperitoneal approach? Eur Urol Focus. (2018) 4(5):632–5. 10.1016/j.euf.2018.08.003 30120075

