# Supplementary material for: Association of PADUA and RENAL scores with early perioperative outcomes in large renal tumors managed with robot-assisted partial nephrectomy
Source: Front Surg. 2026 Apr 30;13:1801634. doi: 10.3389/fsurg.2026.1801634 (PMC13173673; doi:10.3389/fsurg.2026.1801634)
Supplement: Supplementary file 1 [file Table1.docx]

**Supplementary Table S1. Components and definitions of PADUA and RENAL nephrometry scores**

| **Score system** | **Component / domain** | **Scoring categories (points)** | **Definition (imaging-based)** |
| --- | --- | --- | --- |
| PADUA | Tumor size | ≤4 cm (1); 4–7 cm (2); >7 cm (3) | Maximum tumor diameter on cross-sectional imaging. |
| PADUA | Exophytic rate | >50% exophytic (1); 50–0% exophytic (2); entirely endophytic (3) | Proportion of tumor protruding outside renal parenchyma. |
| PADUA | Renal sinus involvement | Absent (1); present (2) | Contact with or extension into the renal sinus. |
| PADUA | Urinary collecting system involvement | Absent (1); present (2) | Contact with or invasion of the collecting system/renal pelvis. |
| PADUA | Longitudinal location (polar lines) | Entirely above/below polar line (1); crosses polar line (2); between polar lines (3) | Location relative to upper and lower renal polar lines. |
| PADUA | Rim location | Lateral (1); medial (2) | Medial tumors are closer to the hilum/sinus and may be more challenging. |
| PADUA | Anterior / posterior | Anterior (a); posterior (p); indeterminate (x) | Suffix describing tumor face; does not change numeric score but aids approach planning. |
| Abbreviations: PADUA, Preoperative Aspects and Dimensions Used for an Anatomical classification | | | |

| **Score system** | **Component / domain** | **Scoring categories (points)** | **Definition (imaging-based)** |
| --- | --- | --- | --- |
| RENAL | R (Radius) | ≤4 cm (1); >4–<7 cm (2); ≥7 cm (3) | Maximum tumor diameter. |
| RENAL | E (Exophytic/endophytic) | ≥50% exophytic (1); <50% exophytic (2); entirely endophytic (3) | Degree of tumor protrusion outside the renal contour. |
| RENAL | N (Nearness) | ≥7 mm (1); 4–6 mm (2); ≤4 mm (3) | Minimum distance from tumor to collecting system or renal sinus. |
| RENAL | A (Anterior/posterior) | Anterior (a); posterior (p); indeterminate (x) | Suffix describing tumor face; does not change numeric score. |
| RENAL | L (Location relative to polar lines) | Entirely above/below polar line (1); crosses polar line (2); >50% between polar lines (3) | Location along the long axis of the kidney. |
| Abbreviations: RENAL, Radius–Exophytic/endophytic–Nearness–Anterior/posterior–Location. | | | |
