# Supplementary material for: Association of PADUA and RENAL scores with early perioperative outcomes in large renal tumors managed with robot-assisted partial nephrectomy
Source: Front Surg. 2026 Apr 30;13:1801634. doi: 10.3389/fsurg.2026.1801634 (PMC13173673; doi:10.3389/fsurg.2026.1801634)
Supplement: Supplementary file 2 [file Table2.docx]

**Supplementary Table S2. Major (Clavien–Dindo ≥IIIa) 30-day postoperative complications by type**

| **Complication type** | **PADUA cohort (n=108)** | | **RENAL cohort (n=83)** | |
| --- | --- | --- | --- | --- |
|  | **n (%)** | **Grade** | **n (%)** | **Grade** |
| Postoperative bleeding | 2 (1.9%) | IIIb | 2 (2.4%) | IIIb |
| Urine leak (collecting system leak/urinary fistula) | 1 (0.9%) | IIIb | 1 (1.2%) | IIIb |
| Pulmonary embolism (PE) | 2 (1.9%) | IVb | 1 (1.2%) | IVb |
| Renal artery pseudoaneurysm (RAP) | 3 (2.8%) | IIIb | 2 (2.4%) | IIIb |
| *Abbreviations:* PE, pulmonary embolism; RAP, renal artery pseudoaneurysm. | | | | |

This table summarizes major complications only (Clavien–Dindo ≥IIIb) within 30 days after RAPN, based on the complication summary provided. Percentages are calculated using the number of patients with available PADUA (n=108) or RENAL (n=83) scores.
